# Supplementary material for: Unpacking the V1 map: Differential covariation of preferred spatial frequency and cortical magnification across spatial dimensions
Source: PLoS Comput Biol. 2025 Oct 27;21(10):e1013599. doi: 10.1371/journal.pcbi.1013599 (PMC12585101; doi:10.1371/journal.pcbi.1013599)
Supplement: S1 Text — (DOCX) [file pcbi.1013599.s009.docx]

### *S1 Text: Three anisotropies in spatial frequency tuning*

Here, we quantified how preferred spatial frequency varied between: 1) the horizontal and vertical meridian of the visual field; 2) radial and tangential stimulus orientations; and 3) horizontal and vertical stimulus orientations.

First, we examined how preferred spatial frequency varied between the horizontal and vertical meridian representation of the visual field, regardless of stimulus orientation. For each observer, we averaged together the preferred spatial frequency measurements for the pinwheels and annuli, with data localized with ±22.5º of either the horizontal or vertical meridian. Averaging across the stimulus classes for each location removes any asymmetry due to radial vs tangential or horizontal vs vertical. Preferred spatial frequency was 20–30% higher along the horizontal than vertical meridian of the visual field, after averaging out stimulus orientation (**FIG S5A**).


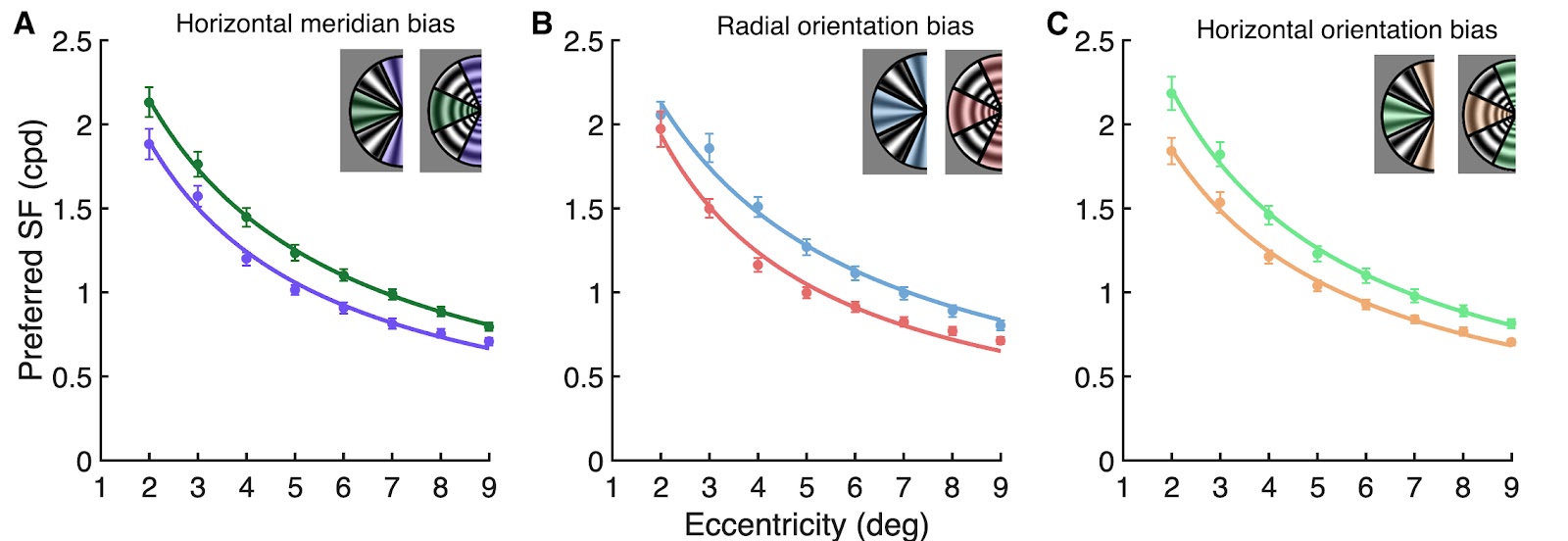


**FIG 1. V1 preferred spatial frequency varies with polar angle meridian and stimulus orientation**. **(A)** Preferred spatial frequency is higher along the horizontal than vertical meridian, after averaging out stimulus orientation. **(B)** Preferred spatial frequency is higher for radial (i.e., pinwheel stimuli) than tangential orientations (i.e., annulus stimuli), when averaged across the polar angle meridians. **(C)** Preferred spatial frequency is higher for horizontal than vertical stimulus orientations, after averaging out polar angle meridian. Error bars represent ±1 SD across 50 bootstrapped group-averages.

Second, we examined how preferred spatial frequency varied between radial and tangential stimulus orientations –irrespective of polar angle meridian. To this end, we computed preferred spatial frequency for the pinwheel (radial) and annulus (tangential) stimuli, averaged across location. For consistency, we restricted the locations to match those for the meridian analysis. Preferred spatial frequency was higher for radial than tangential stimuli, when averaged across the polar angle meridians, with the exception of the 2° eccentricity bin (**S1 Text** **FIG 1B**). Note that averaging across the two meridians not only removes the horizontal vs vertical meridian asymmetry (**S1 Text FIG 1A**), but also any horizontal vs vertical orientation asymmetry (**S1 Text FIG 1C**).

Third, we tested how preferred spatial frequency varied between horizontal and vertical stimulus orientations. To compute preferred spatial frequency for horizontal stimulus orientations, we averaged data from the horizontal meridian for the pinwheels with data from the vertical meridian for the annuli, as both combinations have local horizontal orientation. For vertical stimulus orientations, we did the complement: we averaged data from the vertical meridian from the pinwheels with data from the horizontal meridian from the annuli. Preferred spatial frequency was systematically higher for horizontal than vertical stimulus orientations, after averaging out polar angle meridian (**S1 Text FIG 1C**).

These anisotropies can interact. For example, the choice of stimulus would affect the result if one compared preferred spatial frequency across the different polar angle meridians. In **S1 FIG**, we present measurements of preferred spatial frequency as a function of eccentricity along the horizontal, lower vertical, and upper vertical meridian. When computed from the combined stimuli condition (**S1 FIGA**), preferred spatial frequency is highest along the horizontal, intermediate along the lower vertical, and lowest along the upper vertical meridian of the V1 representation. When computed from the pinwheel stimuli alone (**S1 FIGB**), the polar angle asymmetries increase. When computed from annuli alone (**S1 FIGC**), the polar angle asymmetries decrease.
