# Supplementary figures and images for: Unpacking the V1 map: Differential covariation of preferred spatial frequency and cortical magnification across spatial dimensions
Source: PLoS Comput Biol. 2025 Oct 27;21(10):e1013599. doi: 10.1371/journal.pcbi.1013599 (PMC12585101; doi:10.1371/journal.pcbi.1013599)

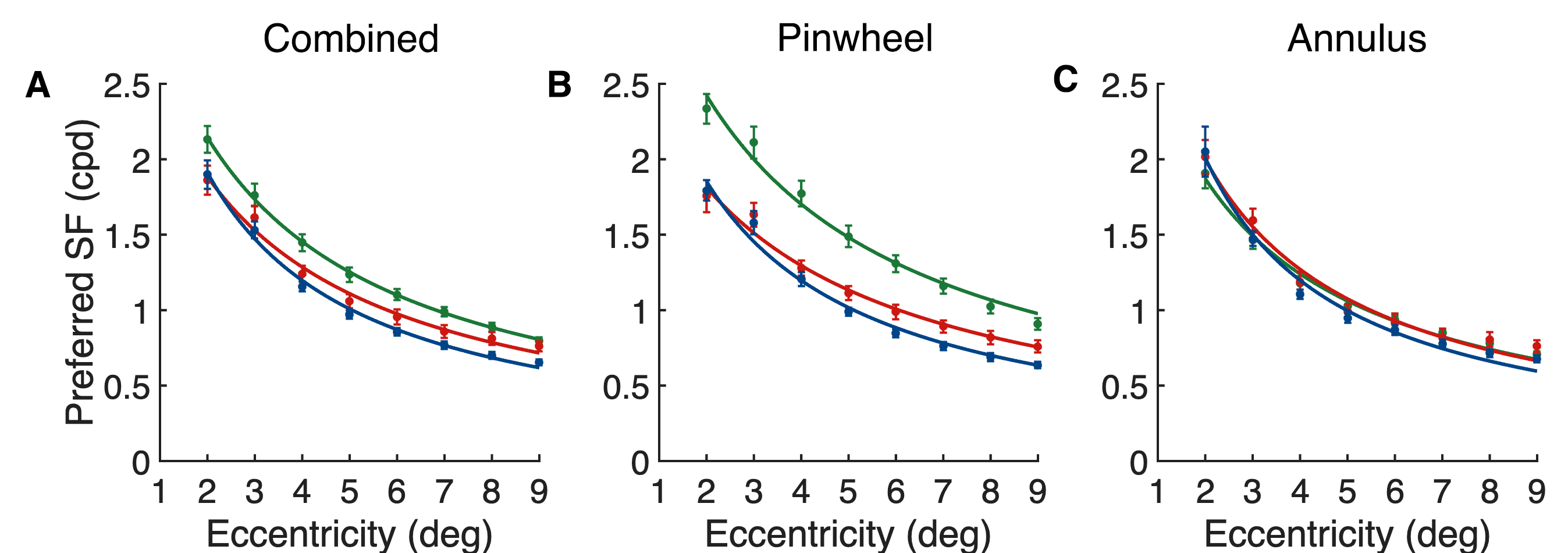

Supplement: S1 Fig — (A) Combined condition; preferred spatial frequency is highest along the horizontal, intermediate along the lower vertical, and lowest along the upper vertical meridian. (B) Pinwheel stimuli; the polar angle asymmetries are boosted as the pinwheel stimuli contain horizontal content along the horizontal meridian and vertical content along the vertical meridian. (C) Annulus stimuli; the polar angle asymmetries are weakened. The data are fit with an inverse linear function from [7]. Error bars represent ±1 standard deviation (SD) across 50 bootstrapped group-averages. (TIF) [file pcbi.1013599.s004.tif]

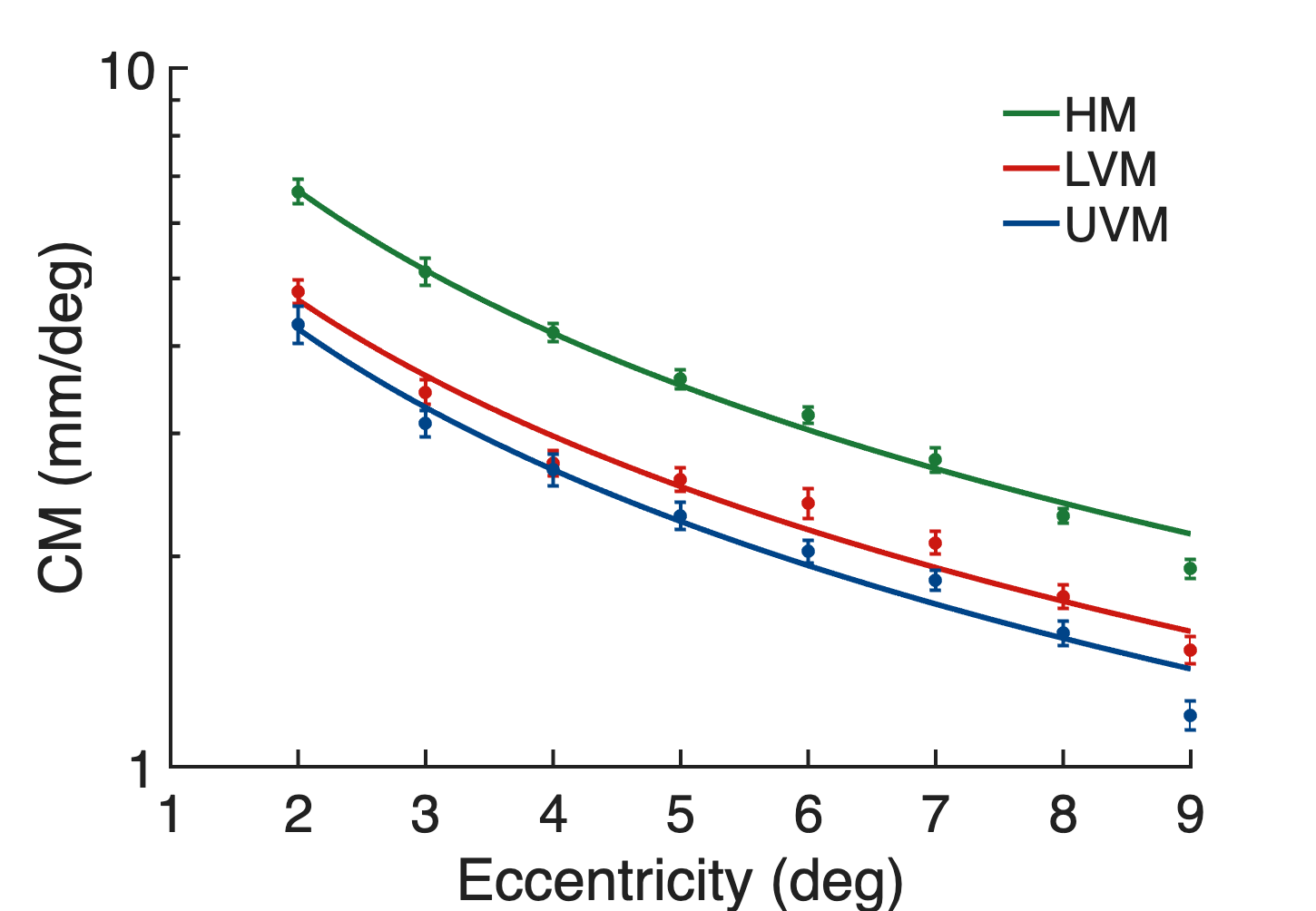

Supplement: S2 Fig — Cortical magnification plotted as a function of eccentricity for the horizontal meridian (HM: average of left and right horizontal), lower vertical (LVM), and upper vertical meridian (UVM). Data come from 22.5° wedge-ROIs centered either side of each meridian. The cortical magnification function from [7] is fit to the data from each meridian. Error bars represent ±1 SD across 50 bootstrapped group-averages. (TIF) [file pcbi.1013599.s005.tif]

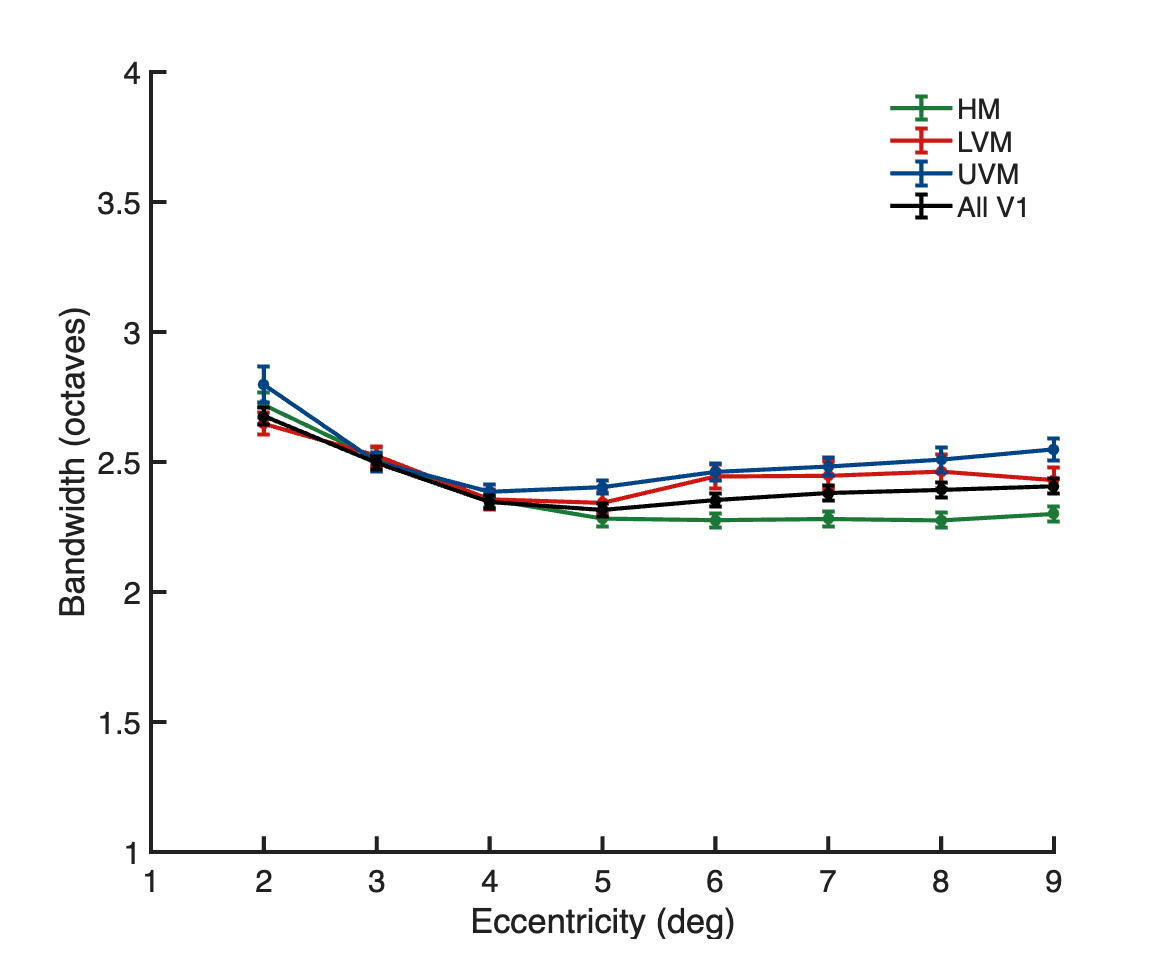

Supplement: S3 Fig — Bandwidth (defined as σ of the log-Gaussian curve) varies as a function of eccentricity when measured along the horizontal, lower vertical, and upper vertical meridian, and all of V1 (combined stimulus condition). The meridian data are derived from 22.5° wedge-ROIs centered on either side of each meridian. All V1 data are averaged around polar angle. Bandwidth is indexed in octaves due to the logarithmic scaling of spatial frequency encoding in the visual system. Error bars represent ±1 standard deviation (SD) across 50 bootstrapped group-averages. (TIF) [file pcbi.1013599.s006.tif]

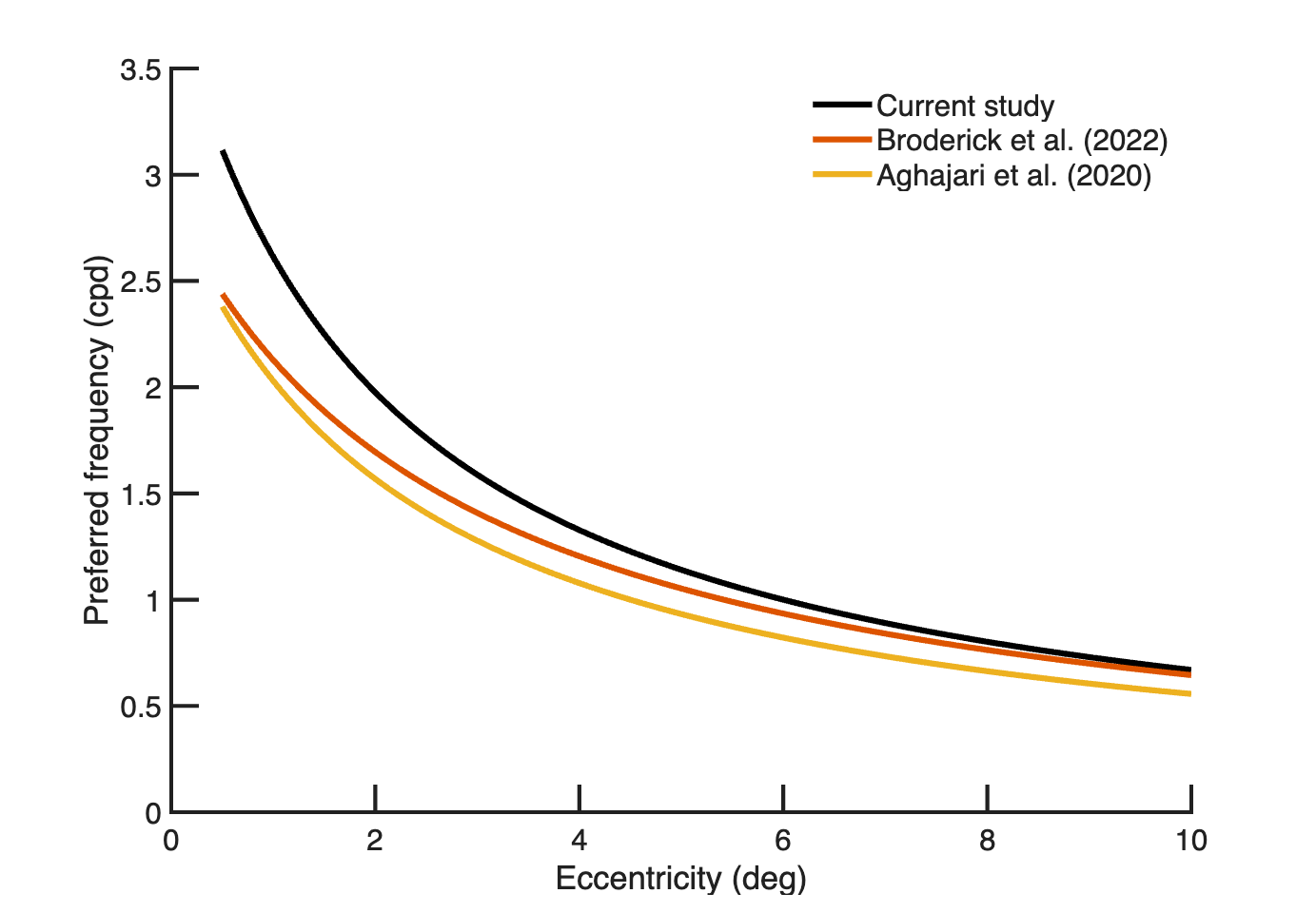

Supplement: S4 Fig — Preferred spatial frequency is plotted as a function of eccentricity from two prior studies and current work. The current study and Broderick et. al. [11] fit the data with an inverse linear function, f(r;=Ar+Bf(r)=Ar+BF(R)=A(R+B). We digitized the V1 data from Aghajari et. al. [12] Fig 4B and fit the same inverse linear function to their data. The three functions have slightly different shapes, however the estimates of preferred spatial frequency are close across the three studies. (TIF) [file pcbi.1013599.s007.tif]

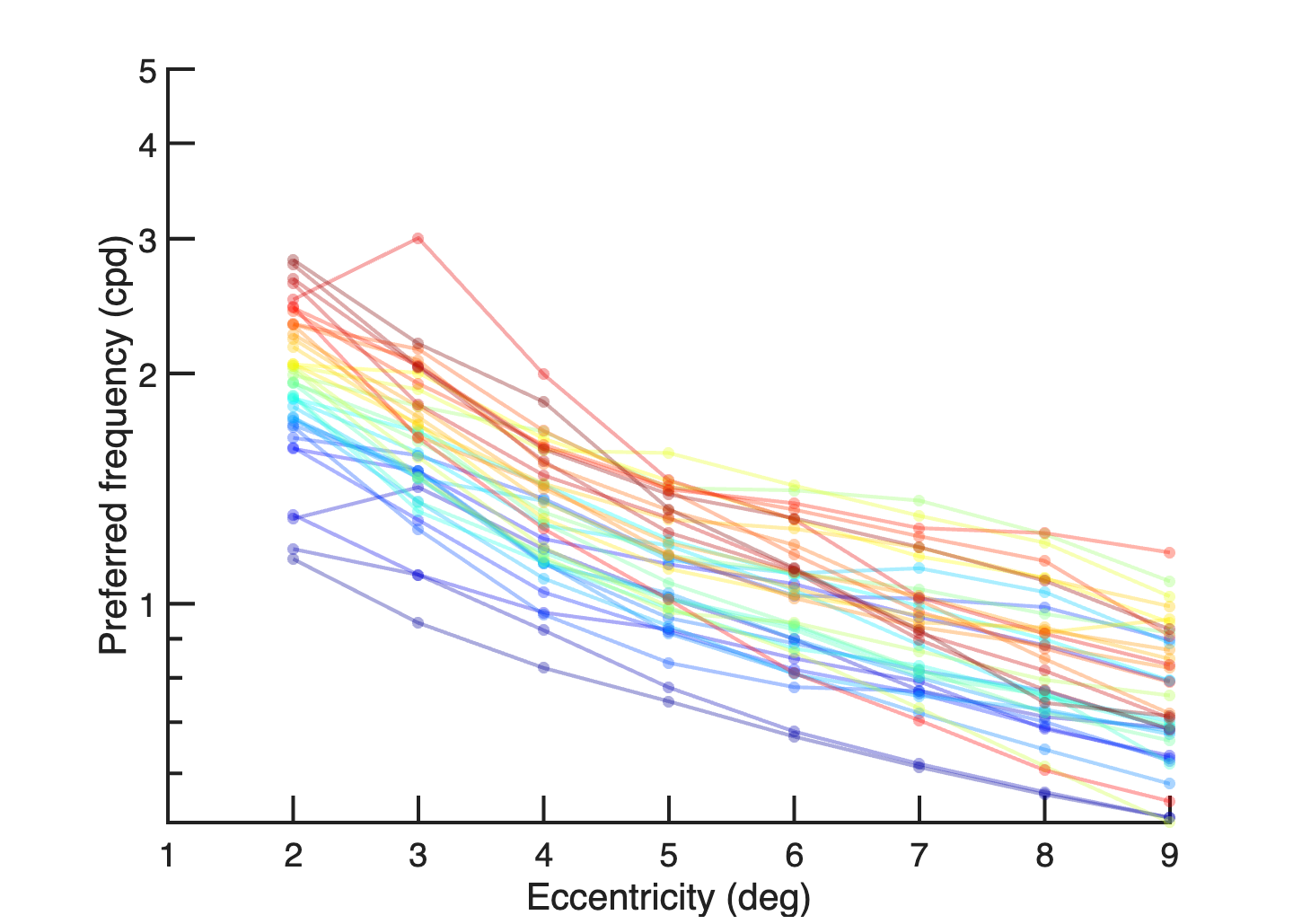

Supplement: S5 Fig — Each colored line represents the change in V1 preferred spatial frequency as a function of eccentricity for an individual observer (n = 40). (TIF) [file pcbi.1013599.s008.tif]
