# Supplementary material for: Unpacking the V1 map: Differential covariation of preferred spatial frequency and cortical magnification across spatial dimensions
Source: PLoS Comput Biol. 2025 Oct 27;21(10):e1013599. doi: 10.1371/journal.pcbi.1013599 (PMC12585101; doi:10.1371/journal.pcbi.1013599)
Supplement: Attachment — Submitted filename: ResponseToReviewers.pdf [file pcbi.1013599.s010.pdf]

**Unpacking the V1 Map: Differential covariation of preferred spatial frequency and cortical magnification across spatial dimensions**

**Response to Reviewers**

We would like to thank the reviewers for their positive and constructive feedback on our manuscript. We have taken their comments into consideration and our responses are below in blue text. Changes to the revised manuscript have been tracked in blue text and are noted below with line numbers for reference. Addressing the reviews has strengthened our manuscript.

---

**Reviewer 1:**

**R1 Q1:** I think the authors should address, head on and early on, the inherent circularity of comparing spatial frequency and cortical magnification, given the stimuli used. Both the radial and the polar gratings varied in spatial frequency with eccentricity, so the spatial frequency driving each voxel was calculated from that voxel's eccentricity. It is no surprise, then, if preferred spatial frequency depends on eccentricity. Similarly, a relationship to cortical magnification is guaranteed by computing spatial frequency after knowing a voxel's eccentricity.

To the degree that different parts of cortex respond independently to different stimuli, one could, in principle, uncover uniform preferred spatial frequency as a function of eccentricity. Hence, we do not think the results are circular. However, we agree with the reviewer that the choice of stimuli (in our case, scaled gratings) likely has some impact on parameter estimation.

**Action:** To assess whether the scaled gratings caused a large bias in the estimation of preferred frequency as a function of eccentricity, we compared preferred spatial frequency across three studies: two with scaled gratings (this paper and and Broderick et. al., 2022) and one with uniform gratings (Aghajari et al., 2020). We find similar estimates of preferred spatial frequency as a function of eccentricity in the three studies. We now report this in the Results (**lines 247-255, page 10**) and in (**Supplementary FIG S4**). We also refer readers to another paper by the senior author, now in review but available as a preprint (Ha et al., 2025, biorxiv). An analysis in this paper shows by simulations that scaled grating stimuli do not bias the model to find a relationship between preferred spatial frequency and eccentricity where none exists.

**R1 Q2:** Fig. 3 caption, line 138 and several other places: how is degrees^2 calculated? I'm assuming it is solid angle -- there really isn't another definition of square degrees, and the numbers look like solid angle numbers. But it's not obvious to someone who hasn't done this kind of work before, and the ms would benefit from 2 or 3 sentences in the Methods specifying that this is solid angle and providing the method for calculating it.

Yes, we compute areal cortical magnification as the ratio of surface area on the brain to solid angle, with surface area measured in $mm^2$ and solid angle in $degrees^2$. We agree that the calculations could be made more explicit. Moreover, for simplicity, we removed areal measures from the manuscript in almost all places and replaced them with linear measures.

**Action**: Added a description of how we calculate solid angle and areal magnification (**lines 857-860, page 26**). We also added scale bars to **FIG 3A and C** to indicate that visual space is measured in deg and cortical space in mm.

**R1 Q3:** The last sentence in the Fig. 5 caption doesn't make sense until you get to the supplemental material and start trying to puzzle out the differences between Fig. 5 and Fig. S4. A few more words would help.

**Action.** We have now removed **FIG S4** and changed other figures that were previously in areal units to be in linear units, with the exception of the bar plot comparing the current measures to prior studies (**FIG 6**), as the prior studies reported cortical magnification in areal units.

**R1 Q4:** In Figs. 7 and 9, the labels to the left of the y-axis label are distracting and might not add to the intelligibility of the figure.

Agreed. We have removed the y-axis labels in **FIG 7**.

We have adjusted y-axis labels and reformatted **FIG 9** for clarity.

**R1 Q5:** Line 314. Is "contrived" supposed to be a different word?

Fixed. (This was supposed to say 'covaried'.)

**R1 Q6:** Line 541: "two" is duplicated

Fixed.

**R1 Q7:** Supp. Fig S1 doesn't really have any context but is intriguing. It would be helpful to have some explanation of why bandwidth was computed and what relationship it has to the findings.

Our primary interest in measuring spatial frequency tuning was to estimate the preferred spatial frequency as a function of eccentricity and polar angle. The reason we computed bandwidth is that it was necessary to estimate the preferred frequency, since bandwidth and preferred frequency are both parameters of the log-Gaussian tuning function. The reviewer is correct that we did not point out the relationship of bandwidth to the findings. In brief, examining the bandwidths enables us to check another aspect of cortical constancies: if cortical units such as hypercolumns are the same up to a scale factor, then spatial frequency bandwidth (in cpd) would scale with preferred spatial frequency, and bandwidth in octaves would be a constant, both as a function of eccentricity and polar angle. We find partial but not complete support for this. We now describe these results and also update Supplementary Fig S3 to show bandwidth as a function of both polar angle and eccentricity.

**Action:** Update to **Supplementary Fig S3** (previously S1) and to results (**lines 233-240, page 9**).

**Reviewer 2:**

**R2 Q1:** In Figure 8, individual differences are assessed globally, i.e., overall V1 surface area vs. overall spatial frequency preference, collapsing across both eccentricity and polar angle. So regional effects may be missed. It would strengthen the study to analyze and/or discuss whether these relationships vary by polar angle.

In our study we fit a 2D model to cortical magnification and preferred spatial frequency at the group-level. To answer the reviewers question, we ran a separate analysis where the models were fit at the individual level. For each observer, we estimated an $\alpha$ and $\beta$ parameter, indexing the HVA and VMA, respectively. We then correlated these values (i.e., does an observer with a larger $\alpha$ for cortical magnification have a larger $\alpha$ for preferred spatial frequency?). Neither parameter was correlated across observers (HVA: $r$ = -0.10; $CI_{95}$ = [-0.50, 0.31], VMA: $r$ = -0.24; $CI_{95}$ = [-0.64, 0.29]).

**Action:** We now report this additional analysis in the results on **lines 413-423, page 15**.

**R2 Q2:** While the link between cortical magnification and spatial frequency is quantified for eccentricity and linked to retinal ganglion cell density (Figure 9), the mechanisms for polar angle variation are less clear. Comparing V1 properties to retinal ganglion cell density across polar angle could clarify whether these asymmetries reflect retinal input or cortical processes.

**Action:** We have amended our discussion to be more explicit regarding the link between retinal ganglion cell density and V1 properties as a function of polar angle (**lines 512-519, page 19**). We have also added some possible reasons as to why V1 cortical magnification amplifies the retinal asymmetries (**lines 527-536, page 19**).

**R2 Q3:** This is entirely optional, but the current title is accurate yet somewhat generic. A more specific title that names cortical magnification, spatial frequency, and the axes of interest (eccentricity, polar angle) would better reflect the paper's content.

**Action:** We have changed the manuscript title to: Unpacking the V1 map: Differential covariation of preferred spatial frequency and cortical magnification across spatial dimensions.

**Reviewer 3:**

**R3 Q1:** In figure 3 the error bars are noted to represent ±1 standard deviation across 50 bootstrapped group-averages -- would it be possible to specify a bit more how the bootstrapping procedure was performed? how many subjects per group etc (this information is I think present later in the manuscript but it would be good to be present here also)

**Lines 129-133, page 6.** The bootstrapping procedure is now first described earlier in the manuscript.

**R3 Q2:** The variation along the polar angle is modeled as a factor that multiplies the average cortical magnification at each eccentricity. Would it be possible to comment whether there are deviations from this assumption? ie how accurate is this assumption?

As noted on **lines 170-172, page 8**, for simplicity the model assumes that variation in polar angle follows the same pattern at every eccentricity (and vice versa). **Supplementary FIG S1A** shows that the change in preferred SF as a function of eccentricity is similar along each polar angle. **Supplementary FIG S2** shows the same pattern for cortical magnification. Thus based on our data, this assumption appears to be a reasonable approximation, although we note that there will inevitably always be some deviation from these assumptions.

**Action:** We updated the text in Results to discuss this issue (**lines 170-172, page 8**).

**R3 Q3:** The authors discuss the implications of their findings for behavioral experiments and suggest that there is good agreement with respect to the variation of spatial frequency/contrast sensitivity as a function of eccentricity. In contrast reports of differences observed along the polar angle are mixed, perhaps because the difference in the typical contrasts of stimuli used for fMRI imaging (high) versus for contrast sensitivity measurements (low). It would strengthen the paper considerably if the authors could implement a psychophysical task to test their observations. However, it is ok with me if this appears in a follow up manuscript.

We agree that strengthening the link between behavior and our neuroimaging data is valuable. This is indeed a priority of ours but doing so is beyond the intended scope of this paper. We believe that the neuroimaging results have value on their own as a basic characterization of the human visual system.
